# Case Report: A Novel Mutation in the CRYGD Gene Causing Congenital Cataract Associated with Nystagmus in a Chinese Family

**DOI:** 10.3389/fgene.2022.824550

**Published:** 2022-02-10

**Authors:** Yunxia Gao, Xiang Ren, Xiangyu Fu, Yu Lin, Lirong Xiao, Xiaoyue Wang, Naihong Yan, Ming Zhang

**Affiliations:** ^1^ Department of Ophthalmology, Ophthalmic Laboratory, West China Hospital, Sichuan University, Chengdu, China; ^2^ Research Laboratory of Ophthalmology and Vision Sciences, State Key Laboratory of Biotherapy, West China Hospital, Sichuan University, Chengdu, China

**Keywords:** congenital cataract, CRYGD gene, AlphaFold2, mutation, nystagmus

## Abstract

**Purpose:** Congenital cataract (CC) is a common disease resulting in leukocoria and the leading cause of blindness in children worldwide. Approximately 50% of congenital cataract is inherited. Our aim is to identify mutations in a Chinese family with congenital cataract.

**Methods:** A four-generation Chinese family diagnosed with congenital cataract was recruited in West China Hospital of Sichuan University. Genomic DNA was extracted from the peripheral blood of these participants. All coding exons and flanking regions were amplified and sequenced, and the variants were validated using Sanger sequencing. AlphaFold2 was used to predict possible protein structural changes in this variant.

**Results:** The proband had congenital nuclear cataract with nystagmus. A heterozygous variant c.233C > T was identified in exon 2 of the *CRYGD* gene in chromosome 2. This mutation resulted in a substitution of serine with phenylalanine at amino acid residue 78 (p.S78F). The variant might result in a less stable structure with a looser loop and broken hydrogen bond predicted by AlphaFold2, and this mutation was co-segregated with the disease phenotype in this family.

**Conclusion:** We described cases of human congenital cataract caused by a novel mutation in the *CRYGD* gene and provided evidence of further phenotypic heterogeneity associated with this variant. Our study further extends the mutation spectrum of the *CRYGD* gene in congenital cataract.

## Introduction

As one of the most remediable causes of visual disability in childhood, congenital cataract is defined as the opacity of the lens onset from birth. The pooled prevalence estimate was 4.24 per 10,000 people, making it a rare disease based on the WHO standards and the highest one is in Asia, which is up to 7.43 per 10,000 individuals (Wu et al., 2016). The opacified lens not only blurs the retinal images but also restrains the development of visual pathways, resulting in visual deprivation amblyopia or nystagmus. Early diagnosis is particularly essential because appropriate and early interventions might contribute to a significant improvement of visual function. However, the visual outcome is largely dependent on the timing of surgery once a dense cataract occurs (Lim et al., 2017). Most vision loss associated with congenital cataract is due to amblyopia (Mansouri et al., 2013), and others are due to postoperative complications such as secondary glaucoma and retinal detachment either from birth or in early infancy (Chak and Rahi, 2008; Koch et al., 2019; Wang et al., 2020). The pathogenesis of congenital cataract can be divided into genetic, environmental, and unknown causes. Inherited congenital cataract, which accounts for up to 50% of congenital cataract, can be mainly classified into the following three different types: autosomal dominant (AD), autosomal recessive (AR), and X-linked recessive (XR). Among them, AD is the most common mode of inheritance, followed by XR (Shiels and Hejtmancik, 2017). Because it is not fatal and would not affect fertility, inherited congenital cataract maintains a relatively high penetrance, and the pathogenic gene can be continuously passed on from generation to generation.

Inherited congenital cataract, which can be inherited as an isolated condition or as a part of other systemic disorders, is mostly caused by genetic mutations, while a few causes refer to chromosomal abnormalities or mitochondrial diseases (Yi et al., 2011). More than half of the cases of congenital cataract are associated with other ocular anomalies such as coloboma (Abouzeid et al., 2009), nystagmus (Qian et al., 2021), strabismus, microcornea (Wang et al., 2013; Leng et al., 2016), or microphthalmia (Jin et al., 2020), persistent hyperplastic primary vitreous (Seitz and Naumann, 1996), congenital glaucoma (Mucke et al., 2004), morning glory syndrome, and persistent pupillary membrane (Song et al., 2014). As a phenotypically and genotypically heterogeneous condition leading to visual loss, the phenotype of inherited cataract broadly reflects spatiotemporal insults experienced by the developing lens. The lens opacities vary from small pulverulent opacities, punctate dots, cortical, anterior polar, posterior polar, lamellar, coralliform, and polymorphic to completely confluent nuclear opacification (Li et al., 2020).

Mutation screening of inherited congenital cataract has identified nearly 200 locus and more than 100 causative genes (Berry et al., 2020), which are well summarized in the “Cat-Map” website (http://cat-map.wustl.edu/) (Santana and Waiswo, 2011). Currently, those reported genes encode different proteins including intracellular lens proteins (α-, β-, and γ-crystallins), water channel proteins, cytoskeletal proteins, transcription factors, and membrane gap junction proteins (Messina-Baas and Cuevas-Covarrubias, 2017). About half of the pathogenic variants occur in the crystallins proteins, which are the major soluble proteins in transparent crystals and are responsible for the development, growth, transparency, and refractive index of the lens (Berry et al., 2020).

In this study, we have undertaken whole-exome sequencing (WES), Sanger sequencing, and bioinformatics analysis to identify and analyze a novel variant in crystallin gamma D (*CRYGD*) gene and further extend the genetic defects of *CRYGD* associated with congenital cataract.

## Methods

### Subjects

In this study, all subjects in the family were identified through the proband attending the ophthalmology department of West China Hospital. This study was performed in accordance with the tenets of the Declaration of Helsinki and was approved by the Ethics Committee of West China Hospital. All individuals taking part in this research have written informed consent. To confirm the affected status and identify whether there were any other ocular abnormalities, all the family members who participated in the study received complete ophthalmic examinations including visual best-corrected acuity (BCVA), intraocular pressure (IOP), slit-lamp biomicroscope, biometry ocular measurement (IOL-Master 700), scanning laser ophthalmoscope (SLO), and optical coherence tomography (OCT).

### Whole-Exome Sequencing

Genomic DNA was extracted from EDTA-sequestered blood samples using the Blood Genome Column Medium Extraction Kit (Kangweishiji, China) according to the manufacturer’s instructions. The extracted DNA samples were subjected to quality control using a Qubit 2.0 fluorimeter and electrophoresis with 0.8% agarose gel for further protocol. The whole-exome library was constructed as reported. Protein-coding exome enrichment was performed using xGen Exome Research Panel v2.0 (IDT, Iowa, USA) that consists of 429,826 individually synthesized and quality-controlled probes, which targets 39 Mb protein-coding region (19,396 genes) of the human genome and covers 51 Mb of end-to-end tiled probe space.

### Sanger Sequencing

Bidirectional direct Sanger sequencing was performed to validate the variant identified by next-generation sequencing. Genomic DNA was amplified by PCR using T3 Super PCR Mix, KAPA2G Robust Hotstart DNA Polymerase (2500 U), and GRYGD-specific primers (forward: 5′-AAA​ACC​CAA​AAT​ATG​CAA​GAG​AGG​A-3′; reverse: 5′-ACT​ATG​AAT​GCA​GCA​GCG​ACC-3′; length 621 bp) designed with Primer3. PCR conditions were as follows: 94°C for 5 min of initial denaturation, followed by 30 cycles of amplification of 30 s at 94°C, 30 s at 60°C, and 45 s at 72°C. High-throughput sequencing was performed by an MGISEQ-T7 series sequencer, and not less than 99% of target sequences were sequenced. The sequencing process was performed by Beijing Chi gene Translational Medicine Research Center Co., Ltd., Beijing.

### Bioinformatics Analysis

Raw data were processed by fastq for adapters removing and low-quality read filtering. The paired-end reads were performed using a Burrows–Wheeler Aligner (BWA) to the Ensemble GRCh37/hg19 reference genome. Base quality score recalibration together with SNP and short indel calling was conducted using GATK. According to the sequence depth and variant quality, SNPs and indels were screened, and high quality and reliable variants were obtained. The online system independently developed by Chi-gene (www.chigene.org) was used to annotate database-based minor allele frequencies (MAFs), and ACMG practice guideline–based pathogenicity of every yielded gene variant, and the system also provides serial software packages for conservative analysis and protein product structure prediction. The databases for MAFs annotation include 1,000 genomes, dbSNP, ESP, ExAC, and Chi-gene in-house MAFs database; Provean, Polypen2, Mutpred, Mutation Taster, SIFT, and Revel software packages were used to predict protein structure variation. As a prioritized pathogenicity annotation to ACMG guideline, OMIM, HGMD, and ClinVar databases were used as conferences of pathogenicity of this variant. The crystallin gamma D (γD-crystallin) amino acid sequences were obtained from the UniProt database (https://www.uniprot.org/). We obtained the structure of wide-type human crystallin gamma D from the Protein Data Bank (PDB) database (ID:2G98). We used the artificial intelligence (AI) software AlphaFold2 to predict the structural changes of crystallin gamma D (Cramer, 2021) and visualized the protein structure using Chimera X software (Download UCSF Chimera).

## Results

### Clinical Features

**FIGURE 1:**
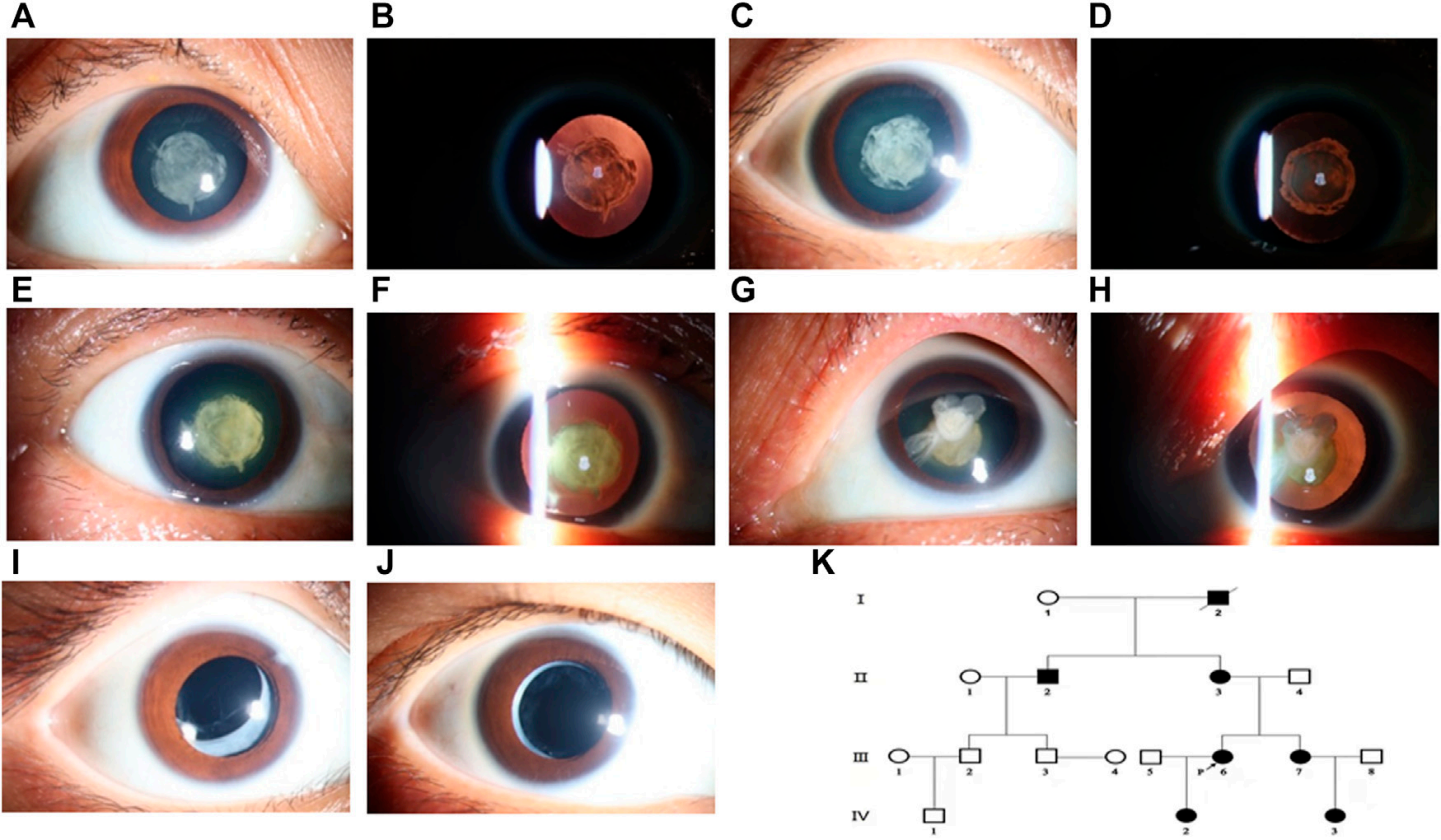
Cataract features and pedigree of the proband and her family members. The phenotype of the proband (III-5) was documented by ophthalmic operating microscope photography and showed a nuclear cataract in diffuse illumination **(A)** and retro-illumination **(B)**. Nuclear opacification **(C**,**D)** was noted for her younger sister (III-7). The same shape of opacification was noted in the both eyes of her mother (II-3), and both nuclear and cortical cataracts were presented **(E**–**H)**. Her daughter (IV-2) and niece (IV-3) also had congenital cataract and received surgery treatment with artificial intraocular lens **(I**,**J)**. Pedigree of the Chinese family with autosomal dominant congenital cataract in four generations **(K)**. Squares and circles represent males and females, respectively. Filled symbols indicate affected members and empty symbols indicate unaffected individuals. The diagonal line indicates a deceased family member, and the arrow indicates the proband.

Members of a four-generation Chinese family affected by autosomal dominant congenital cataract (ADCC) were analyzed from clinical and genetic perspectives. At the evaluation, only the two youngest (III-6 and III-7) had already been submitted to cataract surgery. The history of congenital TORCH infections, ocular trauma, and use of corticosteroids were ruled out. The proband, a 35-year-old female, was diagnosed as bilateral nuclear congenital cataract in both eyes observed by the slit-lamp biomicroscope (Figures 1A,B). The IOL-Master700 revealed that her axial lengths (AL) were 20.53 and 20.86 mm, but the diameter of the cornea was about 11 mm for both eyes with high corneal curvature. Ultrasonography showed no alterations other than lens opacification and a reduced axial length. The best-corrected visual acuity (BVCA) of the proband was 20/200 for both eyes, and no abnormality was found in the retina through SLO. Family history revealed that the proband’s grandfather, sister (Figures 1C,D), mother (Figures 1E–H), uncle, niece, and daughter were diagnosed with bilateral cataract. All the affected individuals showed varying degrees of opacification (rose-like), and their BVCA was less than 20/200 due to amblyopia and exhibited nystagmus as well. Interestingly, the probands’ mother (56-year-old) had both the nuclear and cortical cataract at the same time in the left eye (Figures 1G,H), while her right eye only presented nuclear cataract as the other family members had. Although the two youngest cataract individuals received surgery and intraocular lens implantation (Figures 1I,J) at age of three, visual deprivation amblyopia and severe vision loss still occurred. No systemic diseases or other ocular diseases were found in all affected individuals. Dominant inheritance was supported by the presence of affected individuals in each generation. Ocular abnormities of the affected subjects are summarized in Table 1, and the pedigree of this family with autosomal dominant congenital cataract in four generations is displayed in Figure 1K.

**TABLE 1:** Ocular abnormities in a novel missense mutation in the *CRYGD* gene for congenital cataract.

Subject	Age		Eyes	BVCA	IOP	AL	K1	K2	CCT	ACD	W-W	Ocular abnormalities
I-2	UK	UK		UK	UK	UK	UK	UK	UK	UK	UK	Reported had CC
II-2	UK	UK		UK	UK	UK	UK	UK	UK	UK	UK	Reported had amblyopia and CC
II-3	56	OD		20/200	15.2	NA	NA	NA	NA	NA	NA	NC, nystagmus, amblyopia
		OS		20/200	14	NA	NA	NA	NA	NA	NA	NC, nystagmus, amblyopia
Proband	36	OD		20/200	13.2	20.86	46.05	47.94	552	2.93	11	NC, nystagmus, amblyopia high corneal curvature microphthalmia for both eye
		OS		20/200	11.7	20.53	45.59	47.99	558	2.93	11.11	
III-7	32	OD		20/100	13.8	24.91	44.84	45.44	555	2.77	10.9	NC, nystagmus, amblyopia
		OS		20/100	12.9	24.88	45.28	45.61	550	2.83	10.9	NC, nystagmus, amblyopia
IV-1	9	OD		20/60	10.8	22.13	43.07	44.9	517	NA	10.8	NC, nystagmus, amblyopia
		OS		20/200	11.2	22.18	42.79	45.23	524	NA	10.8	NC, nystagmus, amblyopia
IV-2	15	OD		20/500	10.3	24.1	44.61	46.46	NA	NA	10.8	NC, nystagmus, amblyopia
		OS		20/80	11.5	25.26	45.29	46.6	508	3.7	11.2	NC, nystagmus, amblyopia

BCVA, best-corrected visual acuity; IOP, intraocular pressure; AL, axial lengths; K1, steep anterior corneal curvature; K2, flat anterior corneal curvature; CCT, central corneal thickness; ACD, anterior chamber depth; W-W, white to white for cornea diameter; NC, nuclear cataract; CC, congenital cataract; UK, unknown; NA, not available.

### Bioinformatics Analysis Results

**FIGURE 2:**
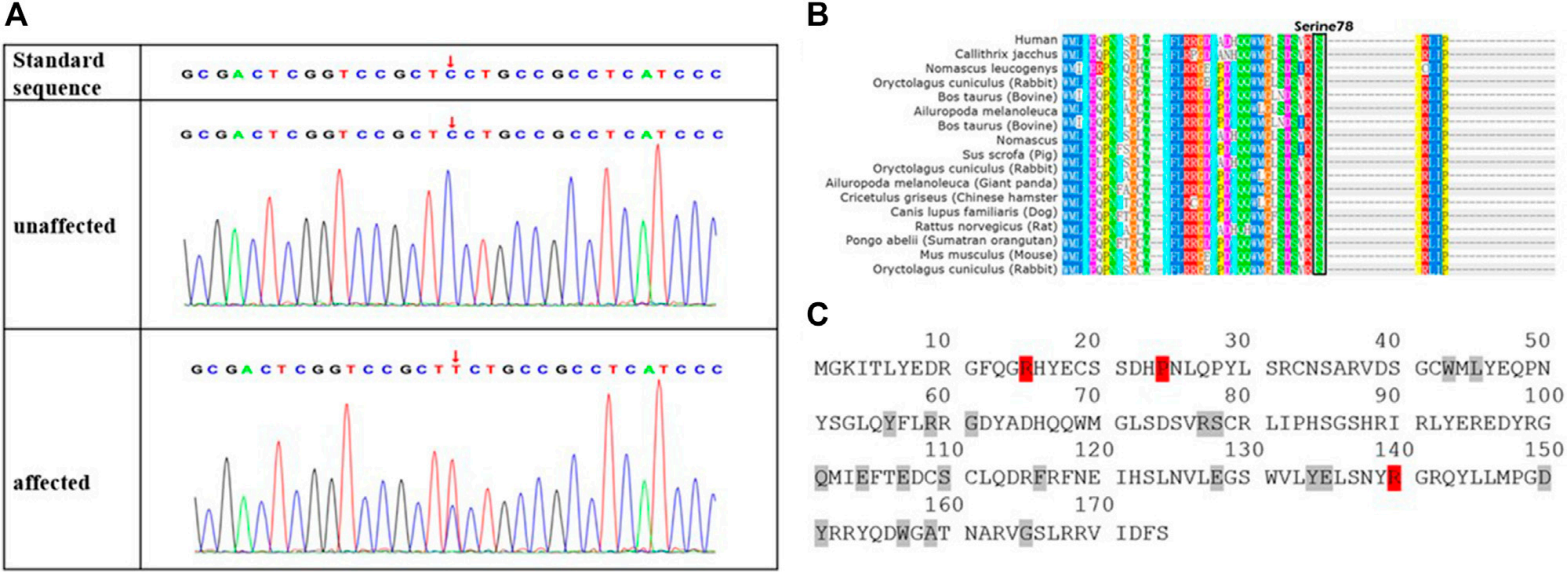
Novel missense mutation (c.233C > T; p.S78F) in *CRYGD* in a Chinese family with congenital cataract. DNA sequences of *CRYGD* in affected and unaffected individuals **(A)**. A heterozygous variant c.233C > T was identified in exon 2 of *CRYGD*. The lower chromatogram of the DNA sequence from the affected proband (III: 6) showed both C and T at the indicated position (red arrows in **A**). The upper sequence chromatogram from an unaffected individual (III: 5) only showed the wild-type *CRYGD* allele. The mutation codon in crystallin gamma D protein is highly conserved in many species, black box indicted the different species had the serine at 78 codon **(B)**, and there are 23 reported mutation residues (with red or gray background) in protein sequence of crystallin gamma D **(C)**. Three (with red background) of these residues are hot spots for mutations, which eventually lead to congenital cataract, while the gray background residues were less frequently mutated.

Next-generation sequencing revealed a novel missense variant. A heterozygous variant c.233C > T was identified in exon 2 of *CRYGD* (Figure 2A). The nucleotide variant resulted in serine-to-phenylalanine substitution at codon 78 (p. Ser78Phe). No previous descriptions had been found in ClinVar, HGMD, UCSC, or Cat-Map. We have registered this novel mutation in ClinVar database, and the accession number is SCV002044295. The serine residue at position 78 is highly conserved across species and isoforms as shown in Figure 2B. The Cat-Map had summarized over 119 mutation gene sites in the human CRYGD protein including 23 variant codon sites (Shiels et al., 2010). The 14th (arginine), 25th (proline), and 150th (arginine) codons were most frequently reported, which are marked in red in Figure 2C. A Spanish author had reported that a missense mutation in c. C232T (p. Ser78Pro) resulted in nuclear cataract (Fernández-Alcalde et al., 2021). Our variant amino acid site (78) was the same as that reported by the Spanish author, but our variant resulted in serine-to-phenylalanine substitution.

**FIGURE 3:**
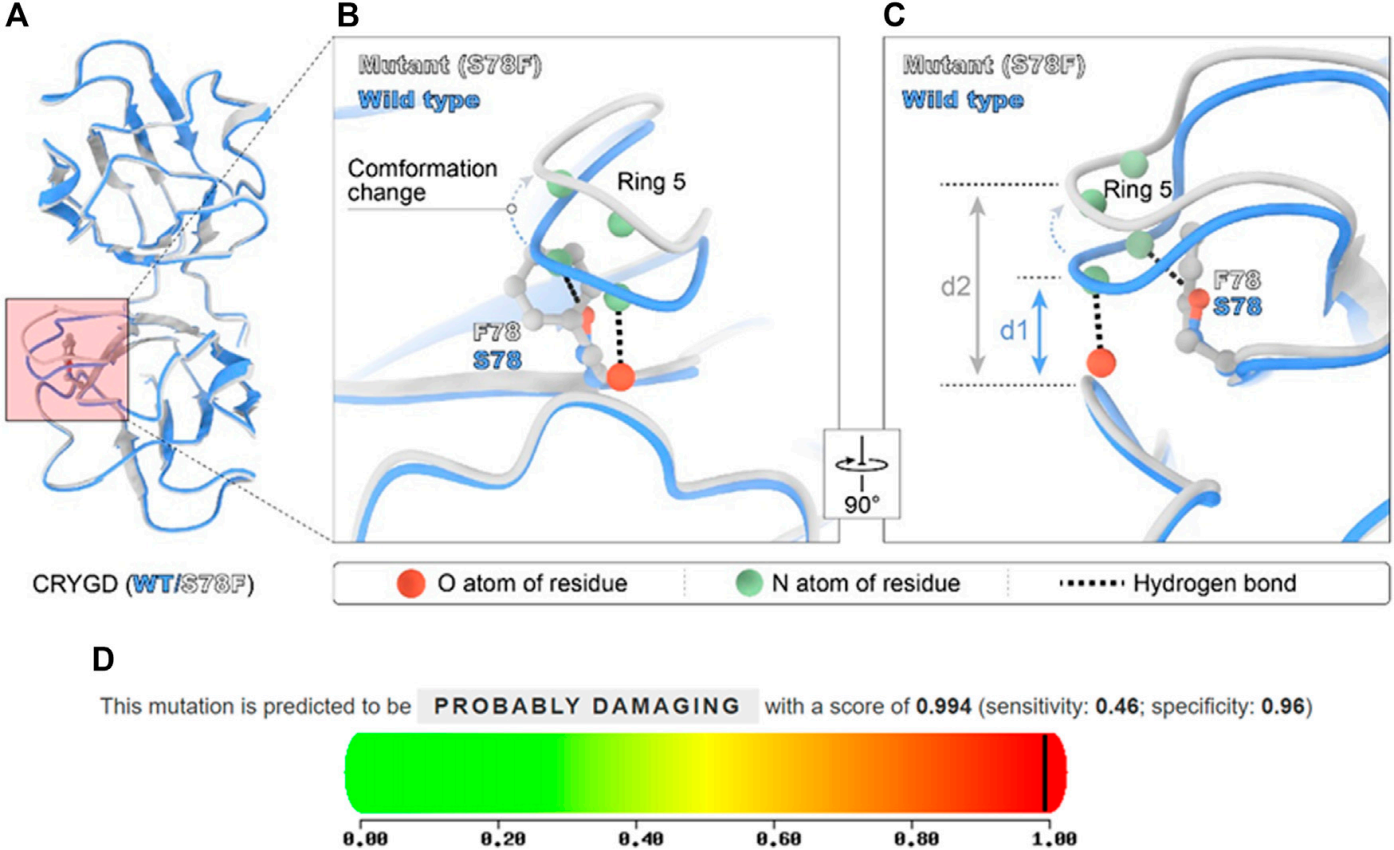
Modeled structure of the CRYGD protein was painted by Chimera X software and the prediction score in PolyPhen-2. Blue indicated CRYGD protein structure in wild type, and the white represent mutated CRYGD protein structure. The mutation described in this study leads to the replacement of serine by phenylalanine at codon 78 (p.S78F), and **(A)** overall structure of CRYGD. **(B)** demonstrates this mutation caused obvious conformation change. The phenylalanine located in the corner of the modeled structure made the fifth ring obvious shifted and longer distance between the two rings. The longer distance broke hydrogen bond between the atoms. **(C)** shows the conformation change by rotating **(B)** 90°, and clearly reveals a longer distance (d1 represents the distance between ring 5 and ring 2 in wild type, and d2 represents the distance between the two rings in the mutated protein) after mutation. **(D)** shows this mutation was predicted as probably damaging in PolyPhen-2 software with a score of 0.994.

Artificial intelligence (AI) technology gradually became an auxiliary means to predict protein structure. In December 2020, the AI company (Deep mind) introduced AlphaFold2 to the world with its extraordinary ability to predict protein structures. AlphaFold2’s database is now an open-source platform for predictions about proteins’ structure with known sequences. Although the overall structure of the S78F mutant was similar to that of the wild type in AlphaFold2 prediction (Figure 3A), the fifth ring around the mutation site was shifted (Figure 3B). The reason for the change was that phenylalanine has a larger side chain than serine and phenylalanine is more hydrophobic than serine. Furthermore, this mutation led to a longer distance between the two rings (Figure 3C). The longer distance not only broke the hydrogen bond interaction between the two rings but also made the local area around S78 residue looser. This local conformational change reduced the stability of the protein and the hydrophobicity of CRYGD. As assessed by the ACMG/AMP 2015 guideline, the p. Ser78Phe alteration in the CRYGD protein would be classified as “likely pathogenic.” The functional impact of the amino acid change was predicted to be damaging based on multiple algorithms by PolyPhen-2 (http://genetics.bwh.harvard.edu), which also predicted this variant as “probably damage” (Figure 3D).

## Discussion

In the present study, we described a Chinese family suffering from congenital cataract in four generations. We undertook the whole-exon sequence to detect functional candidate genes and identified a novel missense mutation (c.233C > T; p.S78F) in CRYGD/γD-crystallin. To date, there are a total of 33 other mutations in 68 articles published that have been identified in *CRYGD*, including 26 missense mutations, two insert mutations, and five small deletions. Almost 90% (61/68) are autosomal dominant inheritance, and 5% are sporadic by pedigree analysis (Li et al., 2020). The most frequently reported missense mutation in GRYGD is c.70C>A, which resulted in a substitution of proline to threonine at codon 24 (p. P24T) (Fan et al., 2020). The substitution of serine to phenylalanine had been reported in congenital cataract caused by variants in gap junction protein alpha 8 (GJA8) protein and crystallin gamma C (CRYGC) (Prokudin et al., 2014), and the mutation in codon 78 (serine) for CRYGD had been reported by a Spanish author. To our knowledge, this pathogenic mutation site (c.233C > T; p.S78F) has not been reported in congenital cataract.

Congenital cataract associated with the CRYGD gene presented different kinds of opacifications, including nuclear, coralliform, punctate dots, cortical, posterior polar, lamellar, coralliform, cerulean, and aculeiform, and the first two are the mostly reported types. Opacities vary in morphology, are often confined to a portion of the lens, and may be static or progressive. All the cataract patients in our study shared nuclear opacification of the lens in the central pupil area with nystagmus and amblyopia. The probands’ mother (56 years old) had both nuclear and cortical cataracts. This cortical opacification is not the same as the senile cataract. And we displayed the varied congenital cataract manifestations in different ages. However, the proband had a decreased AL with a normal-size cornea, while the rest of the affected individuals did not have this phenotype. Moreover, the proband had a relatively high cornea curvature in both eyes. Thus, genetic heterogeneity was presented in this family pedigree. Mutations in *GJA8* (Devi and Vijayalakshmi, 2006), *CRYBB* (Zhou et al., 2010; Jin et al., 2020), *CRYAA* (Liang et al., 2011; Song et al., 2018), and PAX6 (Abouzeid et al., 2009) genes had been widely reported in congenital cataract combined with microphthalmia or microcornea. In our case, the proband did not be diagnosed as microphthalmia as her AL was more than 20 mm. Paolo Capozzi had analyzed biometric and keratometric values in a large population of term-born children with uncomplicated congenital cataract younger than 42 months without microphthalmia, micro- or megalocornea, glaucoma, or retinal disease, and he found the corneal curvature of the eye with the unilateral cataract was significantly greater than that of the eye with a clear lens (Capozzi et al., 2008). The result indicated that the high corneal curvature was not always combined with microphthalmia or microcornea. In addition to cataract, there were three reported mutation sites in CRYGD, resulting in microphthalmia or microcornea (Patel et al., 2016). The S78T mutation in the *CRYGD* gene has been found in a pedigree in Spanish causing nuclear cataract without reduced axial length (Patel et al., 2016). Our pedigree demonstrated that a heterozygous variant c.233C > T resulted in a substitution of serine to phenylalanine at amino acid residue 78 in GRYGD protein and caused nuclear congenital cataract, or high corneal curvature with genetic heterogeneity. This mutation in the *CRYGD* gene might involve the eyeball development, except for the lens. Our study extended genetic mutations and manifestations of inherited cataract related to CRYGD protein. Only the younger two receive surgery treatment at the age of 3 years, and the BCVAs were still damaged. The nuclear cataract put great threat to vision if not found and treated timely in infancy or childhood. Genetic counseling might play an important role in screen and prevention for congenital cataract if diagnosis was made earlier for the proband or subjects.

Crystallins are the most abundant soluble proteins in the ocular lens (80–90%), accounting for optical transparency and a high refractive index of the lens. Since lens central fiber cells lose their nuclei during development, these crystallins are made and then retained throughout life, making them extremely stable proteins. Mammalian crystallins are divided into alpha, beta, and gamma families, and γD-crystallin is a strictly monomeric protein with the lowest molecular mass (20 kDa). γD-crystallin shares the common feature of antiparallel β-sheets in the superfamily protein called the “Greek key motif” (GKM) (Wistow, 2012), and the S78F mutation in the present study is in the second GKM at the N-terminal (Vendra et al., 2013). Serine 78 is in a highly conserved area of *CRYGD* in exon 2, which indicates that the sequence changes in this area play an important role in the onset of congenital cataract. Crystallin proteins expressed with spatiotemporal specify and γD crystallin mainly located in the lens fibers, instead of lens epithelial cells and its mutations usually cause nuclear cataract. Since γD-crystallin expression remains at a relatively high level in the human lens throughout one’s lifetime, the *CRYGD* gene mutation has been associated with congenital cataract, as well as juvenile-onset cataract. In our study, the five affected subjects had almost the same opacity shape, and the older the age, the opaquer were the lens, instead of the enlarged range of the opacity.

We used the well-trained AI software AlphaFold2 to explain the underlying pathogenic mechanism for this variant in CRYGD. The algorithm of AlphaFold2 could accurately predict the 3D structure of a protein based on its amino acid sequences, with an accuracy comparable to that of 3D structures resolved using experimental techniques such as cryo-electron microscopy, nuclear magnetic resonance, or X-ray crystallography. According to the prediction of AlphaFold2, although the overall structure of the S78F mutant was similar to that of the wild type, the fifth ring around the mutation site was obviously shifted. The reason for the change was that phenylalanine has a larger side chain than serine and is more hydrophobic. This local conformational change avoided the clash between atoms caused by the difference between residues, but it led to a longer distance between the two rings. This not only broke the hydrogen bond interaction between the two rings but also made the local area around S78 residue looser. This may decrease the structural stability of the CRYGD protein. With the rapid progress of gene mapping and cloning technology, more and more congenital cataract pathogenic genes have been discovered, which greatly promotes the research on cataract genetics. It is sometimes difficult and time-consuming to conduct molecular biological studies or animal experiments to explain the structure and function of mutated proteins and explore the underlying mechanisms and pathology. Nowadays, AI is widely trained and used in different fields. Computer-aided protein modeling provides a basis for predicting and analyzing the structure of mutated proteins in disease pathogenesis.

In conclusion, we described a pedigree of congenital nuclear cataract caused by a novel mutation in the *CRYGD* gene, which substituted serine phenylalanine at position 78 in the N-terminal region of γD-crystallin. This variant resulted in a less stable structure of CRYGD. Our study also expands the spectrum of known *CRYGD* mutations and provides photographic records of patients with congenital cataract as well as genetic counseling.

## Limitations and Strengths of the Study

We described cases of human congenital cataracts caused by a novel mutation in the CRYGD gene, displayed the spectrum of cataract manifestations, and provided evidence of further phenotypic heterogeneity associated with this variant. Moreover, we used the artificial intelligence to predict and explain the underlying pathogenic mechanism and collected the photographs of cataract in different ages in this four-generation family. One of the limitations of our study is that the molecular mechanism and underlying the pathogenesis of congenital cataract caused by this mutation need to be further investigated and confirmed.

## Data Availability

The datasets for this article are not publicly available due to concerns regarding participant/patient anonymity. Requests to access the datasets should be directed to the corresponding author.
